# The effect of virtual reality and buzzy® on pain, fear, and anxiety during skin prick testing in children: a randomized controlled trial

**DOI:** 10.1007/s00431-026-07020-3

**Published:** 2026-05-05

**Authors:** Funda Gürbüz, Emine Geçkil, Şükrü Nail Güner

**Affiliations:** 1 Vocational School of Health Services, KTO Karatay University, Konya, Turkey; 2 Department of Pediatric Nursing, Necmettin Erbakan University Faculty of Nursing, Konya, Turkey; 3 Department of Pediatrics, Pediatric Immunology and Allergy Division, Necmettin Erbakan University Faculty of Medicine, Konya, Turkey

**Keywords:** Buzzy®, Nursing, Pain, Prick test, Virtual reality

## Abstract

Skin prick testing (SPT), although minimally invasive, is frequently associated with procedural pain, fear, and anxiety in pediatric populations. This randomized controlled trial aimed to compare the effectiveness of immersive virtual reality (VR) and the Buzzy® device in reducing procedural distress during SPT. In this randomized pretest–posttest study, 90 children aged 7–10 years undergoing first-time SPT at a Turkish university hospital were randomly allocated to VR distraction (*n* = 30), Buzzy® (*n* = 30), or routine care (*n* = 30). Pain, fear, and state anxiety were assessed before and after the procedure using validated pediatric scales reported by children, parents, and researchers. Group differences were analyzed using one-way ANOVA and chi-square tests. A mixed-design ANOVA examined group-time effects with Bonferroni-adjusted comparisons. Statistical significance was set at *p* < .05. Groups were comparable at baseline (*p* > .05). Significant group-time effects were identified for pain (F: 27.06, *p* < .001, pη^2^: .38), fear (F: 33.14, *p* < .001, pη^2^: .43), and anxiety (F: 44.76, *p* < .001, pη^2^: .51). Post-procedure scores were lowest in the VR group, followed by Buzzy®, and highest in controls. Effect sizes were large across all outcomes, indicating clinically meaningful reductions in procedural distress. No adverse events occurred.

*Conclusion*: Both VR and Buzzy® were associated with reduced procedural distress during SPT, with VR showing greater reductions than Buzzy® in this study. These findings suggest that structured, nurse-led distraction interventions may be beneficial; however, further studies are needed to confirm these results across different clinical settings. The findings suggest that integrating structured, nurse-led distraction strategies, particularly immersive VR, may substantially enhance atraumatic, child-centered care in pediatric allergy settings. The trial was registered at ClinicalTrials.gov in NCT06443060 (12/04/2025).

**Table:** 

**What is Known:** •*VR and Buzzy® both reduced pain, fear, and anxiety during SPT.* •*Both interventions were safe and feasible in clinical practice.*
**What is New:** •*VR was more effective than Buzzy® in reducing these outcomes.* •*Findings support nurse-led, structured distraction strategies in pediatric allergy settings.*

**What is Known:**

•*VR and Buzzy® both reduced pain, fear, and anxiety during SPT.*

•*Both interventions were safe and feasible in clinical practice.*

**What is New:**

•*VR was more effective than Buzzy® in reducing these outcomes.*

•*Findings support nurse-led, structured distraction strategies in pediatric allergy settings.*

## Introduction

Allergic diseases constitute some of the most prevalent chronic conditions in childhood, affecting 20–40% of individuals globally and imposing a substantial health and economic burden [1–4]. In Western countries, pediatric food allergy rates exceed 8%, and 10% in adults, with projections indicating a continued rise in prevalence in the coming years [3, 4]. Accurate diagnosis is vital for effective management, and skin prick testing (SPT) remains a widely used, reliable, and minimally invasive diagnostic method in pediatric allergy practice. Despite its clinical advantages, SPT may provoke considerable procedural distress, including pain, fear, and anticipatory anxiety in children [5, 6]. Children undergoing SPT and their parents experienced significant levels of procedural pain and anxiety, which were documented, and children’s pain responses were influenced by age and testing conditions [7]. In contrast, parental anxiety contributed to the child’s distress [8]. This finding aligns with broader literature on pediatric needle-related procedures, where pain and anxiety prevalence rates ranging from moderate to high have been repeatedly observed, contributing negatively to children’s immediate experiences and potentially influencing future healthcare interactions. Repeated distressing encounters can amplify anticipatory anxiety, reduce cooperation, and foster negative attitudes toward subsequent medical visits. Furthermore, parental emotional responses, especially maternal anxiety, have been identified as significant predictors of children’s pain perception and procedural distress, indicating that family context plays a critical role in shaping the child’s experience. Given the cumulative effect of procedural anxieties across developmental stages, there is a growing consensus that even minimally invasive procedures deserve targeted attention to minimize psychological sequelae. From a pediatric nursing perspective, minimizing procedural pain, fear, and anxiety is essential to providing atraumatic, family-child-centered care and to fostering positive healthcare experiences early in life [9, 10]. In response to the growing recognition of procedural distress in children [11, 12], non-pharmacological interventions, particularly distraction-based methods, have gained increasing attention in pediatric settings. Virtual reality (VR) offers immersive audiovisual distraction was associated with decreasing pain and anxiety during various procedures [13]. The Buzzy® device combines vibration and cold application, aiming to reduce procedural pain through gate control mechanisms [14]. While both interventions have been associated with benefits in pediatric procedures such as venipuncture and vaccination, evidence regarding their effectiveness during SPT is limited, particularly regarding emotional responses, including fear and anxiety. Additionally, few randomized controlled trials have simultaneously compared different nonpharmacological interventions within the same clinical context, from a nursing care perspective.

Given the high frequency of SPT in pediatric allergy clinics and the central role of nurses in preparing and supporting children during diagnostic procedures, identifying the most effective, feasible, and child-centered nonpharmacological intervention is essential for establishing evidence-based procedural care standards. Therefore, this trial sought to evaluate and compare the efficacy of VR and Buzzy® in reducing pain, fear, and anxiety among children undergoing SPT.

- **H1a:** Children receiving VR or Buzzy® during SPT will report lower pain, fear, and anxiety than those receiving routine care.
- **H1b**: VR will be more effective than Buzzy® in reducing these outcomes.

## Methods

### Study design

**Fig. 1:**
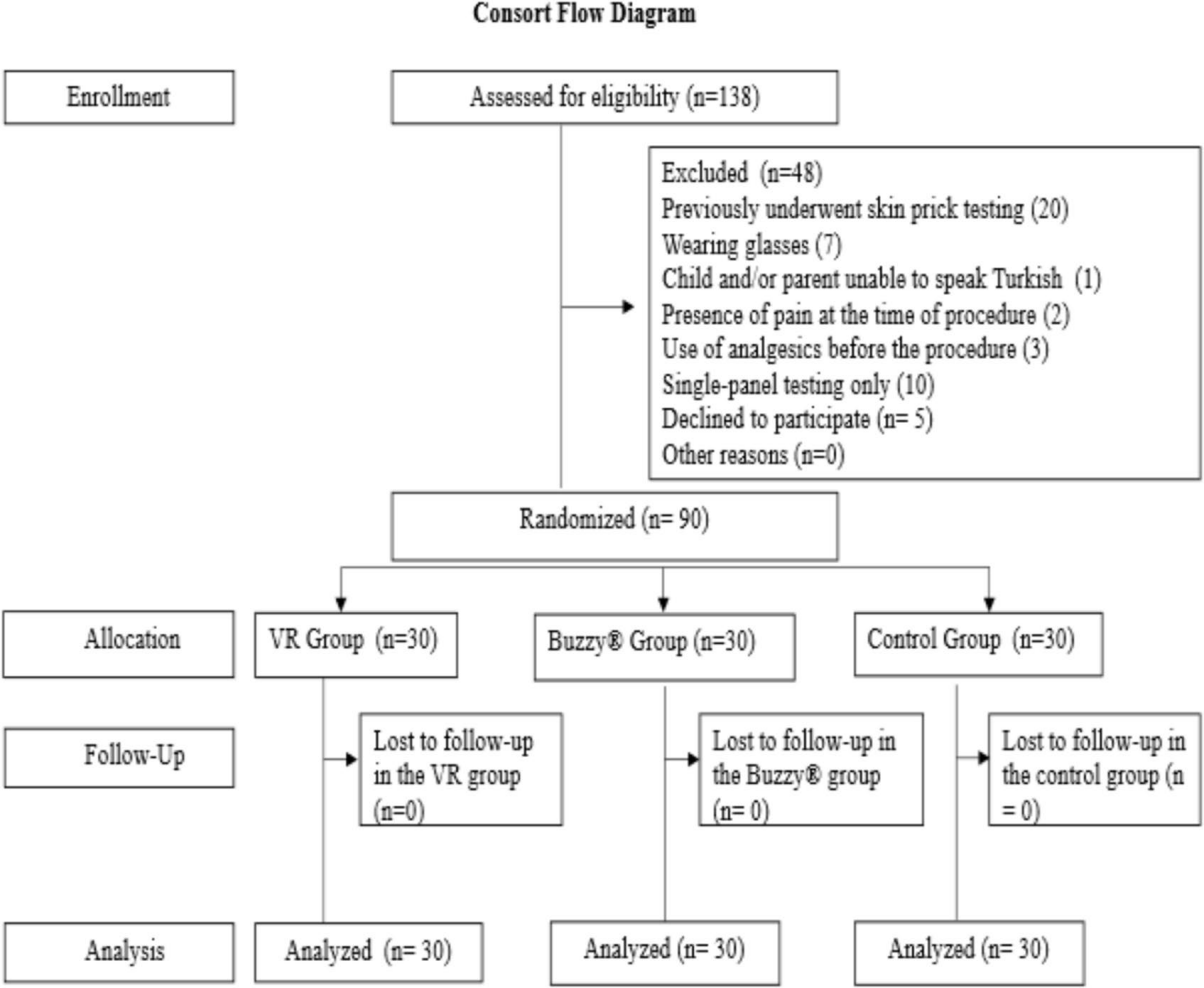
The study’s CONSORT flow diagram (Hopewell et al., 2025)

This study was designed as a parallel-group, randomized controlled trial with a pretest–posttest design to examine the effects of VR and the Buzzy® device on pain, fear, and anxiety in children undergoing SPT. The trial was registered at ClinicalTrials.gov in NCT06443060. The study was conducted and reported in accordance with the Consolidated Standards of Reporting Trials (CONSORT) 2025 Statement: Updated Guideline for Reporting Randomized Trials [15] (Fig. 1).

### Setting and sample

The study was conducted in the pediatric immunology and allergy outpatient clinic of a university hospital in Turkey. The clinic serves children from both urban and rural areas. The sample comprised 90 children aged 7–10 years scheduled for their first SPT. Children were randomly assigned to one of three groups: VR (*n* = 30), Buzzy® (*n* = 30), or control (*n* = 30). Sample size was calculated based on an effect size of 0.16 [16], indicating 90 participants would provide 80% power at α = 0.05.

### Eligibility criteria

Inclusion criteria were children aged 7–10 years, undergoing SPT for the first time using a standardized allergen panel, the ability of both the child and accompanying parent to speak and understand Turkish, and the willingness of both the child and parent to participate.

Exclusion criteria were the presence of visual, hearing, speech, or sensory impairments; chronic illness (other than allergies), undergoing an invasive procedure earlier on the same day; parental conditions preventing behavioral assessment; deformity of the left forearm preventing SPT, presence of pain or use of analgesic medication within the previous six hours [17]. Participants were also excluded if anaphylaxis developed at any stage, the VR or Buzzy® device was removed during the procedure, the test was repeated for any reason, or the child or parent withdrew consent.

### Randomization and blinding

**Fig. 2:**
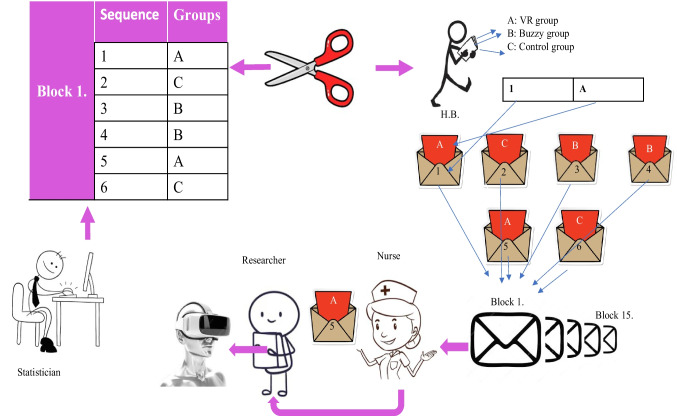
Randomization and blinding *(Visual representation of randomization and blinding prepared by the researcher)*

Participants were assigned to the VR, Buzzy®, or control groups using block randomization with a 1:1:1 allocation ratio. A computer-generated randomization sequence was prepared by an independent statistician using permuted blocks of six. Allocation concealment was ensured through the use of sealed, opaque envelopes prepared by a third party who was not involved in participant recruitment or data collection. Group assignments were revealed only at the time of the procedure by the researcher (Fig. 2). Due to the nature of the interventions, blinding of participants and intervention providers was not feasible. However, standardized and validated outcome measures were used to minimize assessment bias. Data analysis was performed by an independent statistician who was blinded to group allocation to minimize statistical bias.

### Data collection instruments

Demographic characteristics were collected using a researcher-developed information form, including age, sex, and the accompanying parent. Pain was assessed using the Wong–Baker FACES (WB-FACES) Pain Rating Scale, which includes six facial expressions scored from 0 (no pain) to 10 (worst pain) and is suitable for children aged 3–18 years [18]. Fear was measured using the Children’s Fear Scale (CFS), consisting of five facial expressions ranging from 0 (no fear) to 4 (severe fear) and designed for children aged 5–10 years [19]. Anxiety was evaluated using the Children’s Anxiety Scale–State (CAS-D), a thermometer-like visual scale that allows children to indicate their current level of anxiety [19].

### Intervention tools

The VR group viewed a calming 360°, 4 K aquarium video using a head-mounted device, providing immersive distraction. The Buzzy® device, combining cold and vibration, was placed 5–10 cm above the SPT site before and during the procedure. The control group received routine care without additional intervention. Routine care consisted of standard clinical practices in the outpatient setting, including parental presence during the procedure, brief verbal reassurance by the nurse, and a simple explanation of the procedure appropriate to the child’s developmental level. No structured distraction or additional non-pharmacological intervention was provided.

### Procedure

Following the physician’s examination, children and their parents were briefly informed about the SPT process in accordance with routine clinical practice. Baseline pain, fear, and anxiety were assessed immediately before the procedure by the child, parent, and researcher. The children’s pain, fear, and anxiety were assessed both before and immediately after the procedure using the WB-FACES, the CFS, and the CAS-D. All procedures were performed by the same experienced nurse using a standardized protocol in a controlled clinical environment.

Group allocation was determined by sealed-envelope randomization before the procedure. Children in the VR group wore a head-mounted display and viewed a 360° aquarium video beginning approximately one minute before and continuing throughout the procedure. In the Buzzy® group, a device providing simultaneous vibration and cold stimulation was applied 5–10 cm above the test site approximately 5–10 s before allergen application and maintained during the procedure. The timing of the interventions was determined based on prior literature and clinical feasibility. Initiating VR approximately one minute before the procedure allows sufficient time for immersion and attentional engagement, which is critical for effective distraction. In contrast, the Buzzy® device was applied 5–10 s before the procedure in accordance with manufacturer recommendations and previous studies, ensuring activation of the gate control mechanism through simultaneous cold and vibration stimuli at the time of nociceptive input.

Children in the control group received routine care without any additional intervention. No assessments were conducted during the procedure to avoid interference with the intervention or the child’s attention. Immediately after completion of the SPT (within approximately one minute), pain, fear, and anxiety were reassessed independently by the child, parent, and researcher. Following the test, all participants were observed for 15 min in accordance with routine clinical practice.

### Statistical analysis

Data were analyzed using IBM SPSS Statistics (Version 26). Continuous variables were described by means (SD), and categorical variables by frequencies (%). Normality was tested with the Shapiro–Wilk test. Baseline group comparisons for continuous variables were conducted using one-way analysis of variance (ANOVA), while categorical variables were compared using the chi-square test. To evaluate changes in pain, fear, and anxiety scores over time and between groups, a two-way repeated measures analysis of variance (ANOVA) was performed, including group (VR, Buzzy®, control) as the between-subject factor and time (pre- and post-procedure) as the within-subject factor. When significant main or interaction effects were detected, Bonferroni-adjusted post-hoc comparisons were conducted to determine pairwise differences. Effect sizes were reported using partial eta squared (η^2^), interpreted as small (0.01), medium (0.06), and large (0.14). Statistical significance was set at p < 0.05. Post-hoc power analysis indicated a statistical power of 100% for the primary outcome.

## Results

A total of 90 children undergoing SPT were included, randomized into three groups: VR (*n* = 30), Buzzy® (*n* = 30), and control (*n* = 30). Children’s ages, sex distribution, and accompanying parents were similar across groups (*p* > 0.05) (Table 1).

**Table 1:** Comparison of sociodemographic characteristics of children and their parents according to study groups (*N* = 90)

Characteristics	VR (*n* = 30)		Buzzy® (*n* = 30)		Control (*n*:30)		Test	
	*n*	%	*n*	%	*n*	%	χ2	*p*
Child’s gender							1,702	0,427
Female	11	36,7	16	53,3	14	46,7		
Male	19	63,3	14	46,7	16	53,3		
Accompanying parent							3,15	0,487
Mother	21	70	20	66,7	24	80		
Father	9	30	10	33,3	6	20		
Characteristics	**Mean ± SD**	**Median (IQR)**	**Mean ± SD**	**Median (IQR)**	**Mean ± SD**	**Median (IQR)**	**Test F**	**p**
Child’s age (years)	8,60±1,38	8(3)	8,03±1,07	8(2)	8,30 ±1,12	8(2)	1,685	0,191

*χ²* Pearson’s chi-square test, *F* analysis of variance (ANOVA) test statistic, *IQR* interquartile range

Pre-procedure pain scores assessed by children, parents, and the researcher did not differ between groups (*p* > 0.05). However, a significant group-time interaction effect was observed for composite pain scores (F:2.87, pη^2^: 27.06, *p* < 0.001), indicating that changes over time differed across groups. Post-procedure pain scores were significantly lower in the VR group compared with both the Buzzy® and control groups, while the Buzzy® group also reported significantly lower scores than the control group (Bonferroni-adjusted *p* < 0.001). The effect sizes were large (pη^2^: 0.31–0.36), demonstrating clinically meaningful differences between interventions (Table 2).

**Table 2:** Comparison of pre- and post-procedure WB-FACES pain scale scores by group and time and analysis of group–time interaction (*N* = 90)

	Groups		VR group (*n*=30)		Buzzy group (*n*=30)		Control group (*n*=30)		Between-Group Comparison		Group–Time Effect		
	Pain		Mean ± SD	Median (IQR)	Mean ± SD	Median (IQR)	Mean ± SD	Median (IQR)	F (p)	pη^2^		F (p)	pη^2^
Child	Pretest		0 ± 0	0 (<0,001)	0 ± 0	0 (<0,001)	0 ± 0	0 (<0,001)	0,001 (0,999)	0,001	**Group**	**24,028 (<0,001)**	**0,356**
	Posttest		2,60 ± 1,75^a^	2 (2)	4,07 ± 1,34^b^	4 (0,5)	6,07 ± 2,55^c^	6 (4,5)	**24,028(<0,001)**	**0,356**	**Time**	**428,84 (<0,001)** a<b<c*	**0,831**
	Within-group comparison	F(p)	**53,639 (<0,001)**		**131,223 (<0,001)**		**292,034 (<0,001)**				**Group x Time interaction **	**24,028 (<0,001)**	**0,356**
		pη^2^	**0,381**		**0,601**		**0,770**						
Parent	Pretest		0 ± 0	0 (<0,001)	0 ± 0	0 (<0,001)	0 ± 0	0 (<0,001)	0,001 (0,999)	0,001	**Group**	**19,184 (<0,001)**	**0,306**
	Posttest		2,07 ± 1,78^a^	2 (2,5)	4,00 ± 2,92^b^	4 (4)	5,73 ± 2,02^c^	6 (4)	**19,184 (<0,001)**	**0,306**	**Time**	**264,649 (<0,001)** a<b<c*	**0,753**
	Within-group comparison	F(p)	**24,354 (<0,001)**		**91,232 (<0,001)**		**187,431 (<0,001)**				**Group x Time interaction **	**19,184 (<0,001)**	**0,306**
		pη^2^	**0,219**		**0,512**		**0,683**						
Researcher	Pretest		0 ± 0	0 (<0,001)	0 ± 0	0 (<0,001)	0 ± 0	0 (<0,001)	0,001 (0,999)	0,001	**Group**	**22,822 (<0,001)**	**0,344**
	Posttest		2,33 ± 1,40^a^	2 (2)	4,27 ± 2,50^b^	4 (2)	5,87 ± 2,03^c^	6 (4)	**22,822 (<0,001)**	**0,344**	**Time**	**377,691 (<0,001)** a<b<c*	**0,813**
	Within-group comparison	F(p)	**39,693 (<0,001)**		**132,72 (<0,001)**		**250,923 (<0,001)**				**Group x Time interaction **	**22,822 (<0,001)**	**0,344**
		pη^2^	**0,313**		**0,604**		**0,743**						

Mixed-design ANOVA (F) was used for statistical analyses. Effect sizes are presented as partial eta squared (pη²). Descriptive statistics are reported as mean, standard deviation (SD), median (M), and interquartile range (IQR). Bold values indicate statistically significant results (*p* <.001). Bonferroni correction was applied for post hoc comparisons

Similarly, pre-procedure fear scores were comparable among groups (*p* > 0.05). A significant group-time interaction effect was found for fear (*p* < 0.001), with large effect sizes (pη^2^: 0.30–0.31). Post-procedure fear levels were lowest in the VR group, followed by the Buzzy® group, and highest in the control group. Within-group analyses showed significant increases from baseline in all groups; however, the magnitude of increase was smallest in the VR group (Table 3).

**Table 3:** Comparison of pre- and post-procedure children’s Fear Scale (CFS) mean scores by group and time and analysis of group × time interaction (*N* = 90)

	Groups		VR group (*n*=30)		Buzzy group (*n*=30)		Control group (30)		Between-Group Comparison		Group–Time Effect		
	Fear		Mean ± SD	Median (IQR)	Mean ± SD	Median (IQR)	Mean ± SD	Median (IQR)	F (p)	pη^2^		F (p)	pη^2^
Child	Pretest		2,13 ± 0,63	2 (<0,001)	2,07 ± 1,23	2 (2)	2,10 ± 0,84	2 (<0,001)	0,038 (0,963)	0,001	**Group**	**5,59 (0,005)**	**0,114**
	Posttest		1,23 ± 0,43^a^	1 (0,3)	1,70 ± 0,92^b^	2 (1)	2,57 ± 1,04^c^	2 (2)	**19,576 (<0,001)**	**0,310**	**Time**	**8,294 (0,005)** a<b<c*	**0,087**
	Within-group comparison	F (p)	**31,491 (<0,001)**		**5,227 (0,025)**		**8,467 (0,005)**				**Group × Time interaction**	**18,445 (<0,001)**	**0,298**
		pη^2^	**0,266**		**0,057**		**0,089**						
Parent	Pretest		2,10 ± 0,84	2 (1,3)	2,10 ± 1,12	2 (2)	2,00 ± 0,64	2 (<0,001)	0,125 (0,882)	0,003	**Group**	**4,589 (0,013)**	**0,095**
	Posttest		1,20 ± 0,55^a^	1 (1)	1,67 ± 0,71^b^	2 (1)	2,37 ± 0,76^c^	2 (1)	**22,258 (<0,001)**	**0,338**	**Time**	**14,254 (<0,001)** a<b<c*	**0,141**
	Within-group comparison	F (p)	**37,068 (<0,001)**		**8,593 (0,004)**		**6,153 (0,015)**				**Group × Time interaction**	**18,78 (<0,001)**	**0,302**
		pη^2^	**0,299**		**0,090**		**0,066**						
Researcher	Pretest		2,17 ± 0,87	2 (1)	2,13 ± 0,90	2 (1,3)	2,07 ± 0,58	2 (<0,001)	0,122 (0,885)	0,003	**Group**	**4,91 (0,01)**	**0,101**
	Posttest		1,27 ± 0,64^a^	1 (1)	1,87 ± 0,86^b^	2 (1,3)	2,47 ± 0,82^c^	2 (1)	**17,795 (<0,001)**	**0,290**	**Time**	**9,17 (0,003)** a<b<c*	**0,095**
	Within-group comparison	F (p)	**37,91 (<0,001)**		**3,328 (0,072)**		**7,488 (0,008)**				**Group × Time interaction**	**19,778 (<0,001)**	**0,313**
		pη^2^	**0,303**		**0,037**		**0,079**						

Mixed-design ANOVA (F) was used for statistical analyses. Effect sizes are reported as partial eta squared (pη²). Descriptive statistics are presented as mean, standard deviation (SD), median (M), and interquartile range (IQR). Bold values indicate statistically significant results (*p* <.001). Different superscript letters (a, b, c) indicate statistically significant differences between groups based on Bonferroni post hoc test

Anxiety scores before SPT did not differ between groups (*p* > 0.05). Following the procedure, a significant group-time interaction effect was observed for anxiety (*p* < 0.001), with large effect sizes (pη^2^: = 0.41–0.51). Post-procedure anxiety was lowest in the VR group, intermediate in the Buzzy® group, and highest in the control group, indicating that VR produced the strongest attenuation of procedural distress. Post-hoc power analysis confirmed adequate statistical power (100%) for detecting group differences. No adverse events were reported (Table 4).

**Table 4:** Comparison of pre- and post-procedure children’s anxiety scale–state (CAS-D) mean scores by group and time and analysis of group × time interaction (N = 90)

	Groups		VR group (*n*=30)		Buzzy group (*n*=30)		Control group (30)		Between-Group Comparison		Group × Time Effect		
	Anxiety		Mean ± SD	Median (IQR)	Mean ± SD	Median (IQR)	Mean ± SD	Median (IQR)	F (p)	pη^2^		F (p)	pη^2^
Child	Pretest		5,37 ± 1,85	5 (2)	5,27 ± 1,82	5 (2)	5,17 ± 2,67	5 (4)	0,065 (0,937)	0,001	**Group**	**7,251 (0,001)**	**0,143**
	Posttest		1,67 ± 1,15^a^	2 (1)	3,83 ± 2,17^b^	3,5 (3)	5,57 ± 2,80^c^	6 (5)	**24,777 (<0,001)**	**0,363**	**Time**	**52,843 (<0,001)** a<b<c*	**0,378**
	Within-group comparison	F(p)	**96,867 (<0,001)**		**14,537 (<0,001)**		1,132 (0,290)				**Group × Time interaction**	**29,846 (<0,001**	**0,407**
		pη^2^	**0,527**		**0,143**		0,013						
Parent	Pretest		5,03 ± 1,63	5 (2)	5,10 ± 1,49	5 (2)	4,83 ± 1,88	5 (2,3)	0,206 (0,814)	0,005	**Group**	**10,292 (<0,001)**	**0,191**
	Posttest		1,37 ± 1,43^a^	1 (2,3)	3,60 ± 1,71^b^	3 (1,3)	5,07 ± 2,07^c^	5 (3,3)	**33,804 (<0,001)**	**0,437**	**Time**	**95,579 (<0,001)** a<b<c*	**0,523**
	Within-group comparison	F(p)	**158,396 (<0,001)**		**26,508 (<0,001)**		0,641 (0,425)				**Group × Time interaction**	**44,984 (<0,001)**	**0,508**
		pη^2^	**0,645**		**0,234**		0,007						
Researcher	Pretest		4,93 ± 1,55	5 (1,3)	5,40 ± 1,75	5 (2)	5,07 ± 2,30	5 (4)	0,482 (0,619)	0,011	**Group**	**7,022 (0,001)**	**0,139**
	Posttest		1,77 ± 1,28^a^	2 (1,3)	3,43 ± 1,76^b^	3 (2)	4,97 ± 2,37^c^	5 (3,3)	**22,307 (<0,001)**	**0,339**	**Time**	**143,683 (<0,001)** a<b<c*	**0,623**
	Within-group comparison	F(p)	**157,824 (<0,001)**		**60,874 (<0,001)**		0,157 (0,693)				**Group × Time interaction**	**37,586 (<0,001)**	**0,464**
		pη^2^	**0,645**		**0,412**		0,002						

Mixed-design ANOVA (F) was used for statistical analyses. Effect sizes are reported as partial eta squared (pη²). Descriptive statistics are presented as mean, standard deviation (SD), median (M), and interquartile range (IQR). Bold values indicate statistically significant results (*p* <.001). Different superscript letters (a, b, c) indicate statistically significant differences between groups based on Bonferroni post hoc test

## Discussion

This randomized controlled trial, conducted to reduce pain, fear, and anxiety during SPT in children, was associated with the finding that both VR and the Buzzy® device were significantly more effective than routine care, with VR yielding superior outcomes across all measured variables. These findings are clinically meaningful because SPT involves multiple consecutive skin punctures, which may cumulatively intensify children’s procedural distress. Addressing distress during diagnostic procedures is essential not only for immediate comfort but also for preventing long-term healthcare-related fear and avoidance behaviors.

Regarding pain outcomes, both VR and Buzzy® significantly reduced post-procedural pain compared with routine care; however, the magnitude of reduction was greater in the VR group (Table 2). These findings align with studies demonstrating that VR and distraction techniques reduce procedural pain in children [20–23]. Buzzy® also significantly reduced pain compared to the control group, consistent with prior research on IV procedures and vaccinations [24–26]. The procedural characteristics of SPT may explain VR’s superior effect. Unlike single-injection procedures, SPT consists of multiple sequential lancet applications, potentially amplifying pain perception through cumulative nociceptive stimulation and anticipatory mechanisms. Immersive VR likely attenuates this cumulative perception by continuously engaging visual and auditory sensory pathways, thereby limiting attentional resources available for pain processing.

In contrast, Buzzy®, which primarily acts through localized vibration and cold application based on gate control theory, may be less effective during repeated stimuli. The large effect sizes observed suggest that these differences were not only statistically significant but also clinically substantial. This interpretation aligns with previous evidence suggesting that immersive distraction techniques are more effective than localized sensory interventions in reducing procedural pain [27, 28]. Overall, these findings support the study hypothesis that both VR and Buzzy® reduce pain during SPT, with VR demonstrating greater efficacy.

This study found that VR was more effective than Buzzy® in reducing pain during SPT in children. Although no previous studies have directly compared these interventions during SPT, several studies conducted during venipuncture have reported mixed findings. Some studies were associated with the finding that both VR and Buzzy® were effective in reducing pain, with no superiority between methods [19, 29], while others reported no significant benefit compared to control groups [30].

Differences between the present findings and the literature may be explained by methodological variations, pain assessment tools, and procedural characteristics. Unlike venipuncture, SPT involves multiple consecutive painful stimuli, which may intensify pain perception and increase the need for immersive distraction. VR may have been more effective by fully diverting children’s attention from the procedure through continuous audiovisual stimulation, whereas Buzzy®, which provides localized vibration and cold, may have been insufficient to block pain signals during repeated lancet insertions. These findings are consistent with studies suggesting that passive and immersive distraction techniques are more effective than localized or active distraction methods in reducing procedural pain [27, 28]. Overall, the results support the hypothesis that both VR and Buzzy® reduce pain during SPT, with VR demonstrating greater effectiveness.

Fear outcomes were associated with a similar pattern, with VR producing the greatest reduction, followed by Buzzy®, and the highest fear levels observed in the control group. Fear in children undergoing procedures is influenced not only by nociceptive input but also by anticipatory cognitive processes and visual exposure to the procedure (Table 3). This is consistent with the literature showing that immersive distraction techniques, such as VR, can effectively reduce procedural fear in children [31–33]. Previous research has also shown that Buzzy® can reduce fear during invasive procedures such as venipuncture, intramuscular injections, and blood sampling [34–37]. These findings support the notion that Buzzy®, by reducing procedural pain through vibration and cold application, may indirectly alleviate fear responses in children. Although no previous studies have compared VR and Buzzy® specifically during SPT, studies conducted in other invasive procedures have reported inconsistent findings. Some studies identified Buzzy® as the most effective method [38], while others reported no significant difference between VR and Buzzy® [30].

The superiority of VR in reducing fear may stem from its ability to prevent the child from seeing the procedure during the duration of exposure to procedural processing. Children’s fear responses are influenced not only by pain but also by anticipation and cognitive processes. While VR completely distracts attention from the procedure, Buzzy®, which targets physiological pain mechanisms, may have had a limited effect on the emotional components of fear during repeated skin punctures. Additionally, the visual appearance of the Buzzy® device may contribute to fear in some children, as suggested in previous research [26]. These findings support the hypothesis that both interventions reduce fear during SPT, but VR provides a more comprehensive effect.

Similarly, both interventions significantly reduced post-procedural anxiety, with VR showing the strongest anxiolytic effect (Table 4). This finding is consistent with previous studies demonstrating the effectiveness of VR in reducing anxiety during invasive, diagnostic, and perioperative procedures, as well as various pediatric procedures [21, 33, 39–42]. Buzzy® has also been shown to reduce anxiety during needle-related procedures, such as venipuncture, vaccination, and blood sampling by attenuating pain-related physiological responses [24, 27, 36]. However, in the present study, Buzzy® was less effective than VR in reducing anxiety. The observation of a greater anxiolytic effect of VR in this study may be related to the nature of SPT, which involves repeated painful stimuli and can increase anticipatory anxiety due to predictable pain. While Buzzy® may reduce anxiety indirectly through pain attenuation, VR appears to engage both sensory and cognitive pathways, offering a more comprehensive modulation of emotional distress. This interpretation is consistent with evidence showing a bidirectional relationship between pain and anxiety; this relationship implies that a reduction in pain contributes to a reduction in anxiety [43]. Overall, the findings of this study indicate that both VR and Buzzy® may help reduce procedural pain, fear, and anxiety in children undergoing SPT, with VR demonstrating greater effectiveness. These results are consistent with existing literature on distraction-based interventions; however, differences in study design, population, and procedural characteristics should be considered when interpreting the findings.

From a clinical perspective, these findings underscore the importance of integrating evidence-based, nurse-led distraction strategies into pediatric allergy clinics. Pediatric nurses are uniquely positioned to assess children’s emotional readiness, select appropriate non-pharmacological interventions, and implement strategies that enhance procedural cooperation and comfort. Although VR was associated with superior effectiveness, practical considerations such as equipment cost, infection control procedures, staff training, and workflow integration should be considered when implementing such technologies in routine outpatient settings. Collectively, this study supports the incorporation of structured distraction interventions during SPT to reduce procedural pain and emotional distress. By systematically addressing both sensory and psychological components of procedural experiences, pediatric nurses can enhance immediate comfort, strengthen family trust, and potentially influence children’s long-term attitudes toward healthcare encounters.

### Practice implications

The findings of this study have important implications for pediatric nursing practice in allergy clinics. Both VR and the Buzzy® device may be considered as supportive non-pharmacological interventions during SPT to help reduce procedural distress in children, thereby supporting atraumatic and family-child-centered care. Given its superior effectiveness, VR may be particularly beneficial for children experiencing high levels of anxiety or undergoing their first invasive procedure. However, implementation decisions should be individualized based on clinical resources and patient needs. Pediatric nurses play a key role in assessing children’s emotional needs, selecting appropriate non-pharmacological interventions, and implementing strategies that enhance children’s comfort and cooperation during diagnostic procedures. Incorporating these interventions into standard nursing protocols may improve procedural experiences for children and families, increase satisfaction with care, and promote positive long-term attitudes toward healthcare.

### Strengths and limitations

This study has several strengths, including its randomized controlled design, the use of validated outcome measures, and the comprehensive assessment of pain, fear, and anxiety from multiple perspectives (child, parent, and researcher). However, some limitations should be acknowledged. The study was conducted in a single center, which may limit the generalizability of the findings. In addition, the definition of routine care may vary across clinical settings, potentially influencing comparisons between groups. Blinding of participants and intervention providers was not feasible due to the nature of the interventions, which may have introduced performance bias. Furthermore, outcomes were assessed only immediately after the procedure, preventing evaluation of long-term psychological effects. Future studies should include multicenter designs, broader age groups, repeated procedures, and longer follow-up periods to confirm and extend these findings.

## Conclusions

Both VR and Buzzy® were associated with reductions in pain, fear, and anxiety during SPT in children compared with routine care. VR demonstrated superior effectiveness across all measured outcomes, suggesting that immersive distraction may represent a more comprehensive approach to managing procedural distress. However, these findings should be interpreted in light of the study’s limitations, including the single-center design and lack of blinding. Further multicenter studies with larger samples and longer follow-up periods are needed to confirm these findings and to evaluate their long-term impact on children’s healthcare experiences.

## Data Availability

The dataset for this study will be shared by the author if needed.
